# Biocontrol Potential, Plant Growth-Promotion, and Genomic Insights of *Pseudomonas koreensis* CHHM-1 Against Bacterial Canker in *Actinidia arguta*

**DOI:** 10.3390/microorganisms13102400

**Published:** 2025-10-20

**Authors:** Mengqi Wang, Taiping Tian, Yue Wang, Ruoqi Liu, Shutian Fan, Mingjie Ma, Baoxiang Zhang, Jiaqi Li, Yanli Wang, Yiming Yang, Peilei Xu, Nan Shu, Wenpeng Lu, Bowei Sun, Manyu Wu, Hongyan Qin, Changyu Li

**Affiliations:** 1 Institute of Special Animal and Plant Sciences, Chinese Academy of Agricultural Sciences, Changchun 130112, China; 82101232246@caas.cn (M.W.); 82101235243@caas.cn (T.T.); wangyue05@caas.cn (Y.W.); 821012450308@caas.cn (R.L.); fanshutian@caas.cn (S.F.); zhangbaoxiang@caas.cn (B.Z.); lijiaqi@caas.cn (J.L.); wangyanli@caas.cn (Y.W.); yangyiming@caas.cn (Y.Y.); xupeilei@caas.cn (P.X.); shunan@caas.cn (N.S.); luwenpeng@caas.cn (W.L.); sunbowei@caas.cn (B.S.); wumanyu@caas.cn (M.W.); 2 College of Agriculture, Yanbian University, Yanji 133002, China; 2023050869@ybu.edu.cn

**Keywords:** *Pseudomonas syringae* pv. *actinidiae*, rhizosphere isolate, antimicrobial activity, sustainable agriculture, disease management

## Abstract

In 2019, bacterial canker caused by *Pseudomonas syringae* pv. *actinidiae* was first identified in *Actinidia arguta*. This disease has led to significant yield reduction, plant mortality, and substantial economic losses in *A. arguta* cultivation. Its emergence poses a novel challenge to the sustainable global production of kiwifruit. Currently available treatments for bacterial canker caused by *P. syringae* pv. *actinidiae* are scarce. Moreover, the environmental toxicity of copper-based compounds and emerging antibiotic resistance issues necessitate the development of eco-friendly control strategies. Disease management strategies based on biocontrol bacteria have shown broad application prospects. In this study, the isolate CHHM-1 with significant antagonistic activity against *P. syringae* pv. *actinidiae* was isolated from the rhizosphere soil of healthy *A. arguta*. It was identified as *Pseudomonas koreensis* through 16S rRNA gene and whole-genome sequencing. Genomic analysis revealed that the isolate CHHM-1 harbors various genes related to biocontrol, plant growth promotion, and antibiotic resistance, suggesting strong environmental adaptability and functional potential. Furthermore, the strain exhibited multiple plant growth-promoting traits, such as nitrogen fixation, phosphate solubilization, siderophore production, and synthesis of indole-3-acetic acid (IAA). In vitro antagonism assays confirmed the strong antagonistic activity of the isolate CHHM-1 against *P. syringae* pv. *actinidiae*. A dual-culture plate assay showed an average inhibition zone of 4.36 cm, while preventive application on plants significantly reduced lesion length to 1.3 mm (vs. 6.2 mm control) in shoots and lesion area to 10% (vs. 80% control) in leaf discs. Further antibacterial tests revealed that its inhibitory mechanism is attributed to secreted antimicrobial substances. These findings provide a promising candidate for developing novel biopesticides to combat *P. syringae* pv. *actinidiae* variants, reduce chemical dependency, and foster sustainable *A. arguta* production.

## 1. Introduction

*Actinidia arguta* (Sieb. & Zucc) Planch. ex Miq., commonly known as Ruan-Zao-Zi, is a large deciduous vine of the genus *Actinidia* in the *Actinidiaceae* family. As an emerging specialty fruit crop, *A. arguta* produces small fruits characterized by a smooth, edible peel and a distinctive aromatic profile [1]. These fruits exhibit higher antioxidant capacity compared to fuzzy kiwifruit (*A. deliciosa*), a feature particularly notable in their thin, uniquely flavoured skin that enables complete consumption without peeling [1]. The cultivation of *A. arguta* has evolved into a large-scale industry in China’s three northeastern provinces, with a total planted area of 3000 hectares [2,3,4]. Nevertheless, the sustainable development of this industry is confronting severe challenges.

In 2019, bacterial canker was first observed in *A. arguta* in China [5]. The disease can invade multiple organs, including trunks, branches, young shoots, leaves, and flower buds. Typical symptoms include the exudation of rust-colored sap from branches and the appearance of necrotic lesions surrounded by chlorotic halos on leaves [5,6,7]. In previous work, the isolation and identification of the pathogen causing bacterial canker in *A. arguta* ‘Longcheng No. 2’ was reported through morphological characterization and sequence analysis with specific primers [5]. The pathogen, *Pseudomonas syringae* pv. *actinidiae* biovar 2, belongs to a different subgroup than the predominant *P. syringae* pv. *actinidiae* biovar 3, which is responsible for kiwifruit canker in *A. chinensis* and *A. deliciosa*.

*P. syringae* pv. *actinidiae* invades host plants through natural openings and wounds via flagellar motility [8]. The bacterial biofilm enhances resistance to host defenses and antimicrobial agents. This disease has become the most devastating threat to *A. arguta* cultivation [5]. It often causes large-scale tree mortality within short periods, resulting in severe damage to the *A. arguta* industry in China. Current disease management relies primarily on preventive measures due to the lack of effective curative treatments against *P. syringae* pv. *actinidiae,* with approved control agents being mainly copper-based compounds and antibiotics [7]. However, copper formulations exhibit significant phytotoxicity, and both control strategies contribute to the development of *P. syringae* pv. *actinidiae* resistance. Consequently, developing sustainable agricultural strategies to prevent the emergence of resistant *P. syringae* pv. *actinidiae* strains has become a critical research priority.

In recent years, beneficial microorganisms have received increasing attention due to their significant advantages in agricultural safety, economy, and sustainability over traditional fungicides. Consequently, disease management strategies based on biocontrol bacteria have shown broad application prospects [9]. Biocontrol bacteria suppress pathogens through multiple mechanisms, including direct antagonism and indirect modulation of plant defenses. These involve hyperparasitism, nutrient competition, production of antibiotic substances, and induction of systemic resistance [10]. For example, *P*. *fluorescens* produces antimicrobial metabolites such as 2,4-diacetylphloroglucinol (2,4-DAPG) and phenazines [11], which exhibit potent inhibitory effects against a wide range of phytopathogens. However, effective biocontrol resources targeting *P. syringae* pv. *actinidiae* biovar 2, the pathogen causing bacterial canker in *A. arguta*, remain relatively scarce.

This study successfully isolated a promising rhizobacterial strain, CHHM-1, which was identified as *Pseudomonas koreensis*. This strain not only significantly inhibits the growth of *P. syringae* pv. *actinidiae* but also possesses multiple plant growth-promoting traits, including nitrogen fixation, phosphate solubilization, siderophore production, and synthesis of indole-3-acetic acid (IAA). Whole-genome analysis revealed its rich functional gene repertoire and intraspecific genetic diversity.

In summary, this study is the first to report the significant potential of *P. koreensis* CHHM-1 in controlling bacterial canker in *A. arguta*. By integrating both biocontrol and growth-promoting functions, this strain provides an excellent candidate and a solid theoretical foundation for developing novel multifunctional microbial agents against this devastating disease, highlighting its critical importance for promoting green and sustainable development of the *A. arguta* industry.

## 2. Materials and Methods

### 2.1. Test Materials

*P. syringae* pv. *actinidiae* strain R12 was originally isolated and identified from *A. arguta* exhibiting symptoms of bacterial canker in Liaoning Province, China, in 2019 by the Special Wild Economic Animal and Plant Research Institute, Chinese Academy of Agricultural Sciences [5].

Ten rhizosphere soil samples were collected on 7 November 2024, under ten different, apparently healthy *A. arguta* plants in an orchard affected by bacterial canker in Yangmuchuan Town, Kuandian Manchu Autonomous County, Dandong City, Liaoning Province, China (GPS coordinates: 40° N, 124° E). The samples were obtained from soil adhering to roots within a 1 mm distance.

### 2.2. Isolation and Purification of Rhizosphere Bacteria

Rhizosphere bacteria were isolated using the serial dilution method. Briefly, 5 g of soil was added to a 250 mL conical flask containing 100 mL of sterile water. The suspension was incubated at 28 °C with shaking at 200 rpm for 1 h and allowed to settle. Subsequently, 100 μL of the supernatant was subjected to a series of ten-fold dilutions in sterile water, generating dilution gradients ranging from 10^−1^ to 10^−7^. Aliquots (100 μL) from the appropriate dilutions were spread onto Nutrient Agar (NA, Hope Bio, Qingdao, China) medium, with three replicate plates prepared per dilution. All plates were incubated at 28 °C for 3 days. Colonies with distinct morphologies were selected, subcultured for purification, and stored at −80 °C in an ultrafreezer for long-term preservation.

### 2.3. Screening of Antagonistic Bacteria

A dual-culture plate assay was performed with *P. syringae* pv. *actinidiae* as the indicator strain. The surface of the NA medium was inoculated with 200 μL of a 10^4^-fold diluted *P. syringae* pv. *actinidiae* suspension (~7 × 10^7^ CFU/mL). A sterile 9 mm filter paper disc, to which 10 μL of the antagonist culture (1 × 10^8^ CFU/mL) had been applied, was placed centrally. The experimental design included two independent trials with three replicates each. After incubation at 28 °C for 7 days, the inhibition zones were measured.

### 2.4. Identification of the Antagonistic Isolate CHHM-1

The antagonistic bacteria were identified through morphological observation and phylogenetic analysis. For colony morphology observation, the isolate CHHM-1 was grown on NA medium at 28 °C for 48 h. Cellular morphology was examined using Gram staining. Physiological and biochemical characteristics were determined using Hope bio (Qingdao, China) commercial identification kits, with reference to Bergey’s Manual of Systematic Bacteriology and the Handbook of Systematic Identification of Common Bacteria. The tests included oxidase activity, starch hydrolysis, Voges–Proskauer (V-P) test, gelatin liquefaction, nitrate reduction, hydrogen sulfide production, indole production, growth at pH 5.7, urease activity, and glucose utilization.

The selected isolate CHHM-1 was inoculated into Luria–Bertani (LB, Hope Bio, Qingdao, China) liquid medium and grown at 28 °C with shaking at 120 r/min, reaching the late log phase following 12 h of growth. The sample was subsequently analyzed using scanning electron microscopy (SEM) to assess bacterial morphology as described in Section 2.12.

The selected isolate CHHM-1 was inoculated into LB medium and grown at 28 °C with shaking at 120 r/min, reaching the late log phase following 12 h of growth. Bacterial cells were collected and sent to Majorbio Biotechnology (Shanghai, China). for sequencing. The 16S rRNA gene was amplified using universal primers 27F and 1492R [12]. Amplification was performed on an ABI GeneAmp 9700 thermocycler (Thermo Fisher, Singapore, Singapore) with the following program: initial denaturation at 95 °C for 3 min; 35 cycles of denaturation at 95 °C for 30 s, annealing at 55 °C for 30 s, and extension at 72 °C for 45 s; and final extension at 72 °C for 10 min, followed by a hold at 10 °C. The PCR products were purified, bidirectionally sequenced, and assembled, yielding a 1448 bp sequence of the target gene. This sequence was compared against the NCBI database (https://blast.ncbi.nlm.nih.gov/Blast.cgi, accessed on 10 May 2025) using BLAST. Related sequences were downloaded for multiple sequence alignment, and phylogenetic analysis was performed using MEGA 7.0 software. Phylogenetic trees were constructed using the Maximum Likelihood and Neighbor-Joining methods [13], with bootstrap values based on 1000 replicates.

### 2.5. Whole-Genome Sequencing and Analysis

The isolate CHHM-1 was grown in LB medium at 28 °C with shaking at 120 r/min, reaching the late log phase following 12 h of growth. A 50 mL aliquot of bacterial culture was sent to Majorbio for genome sequencing.

The complete genome of the isolate CHHM-1 was sequenced using a hybrid approach combining second-generation (Illumina, San Diego, CA, USA) and third-generation (PacBio, Menlo Park, CA, USA) technologies. Sequencing generated at least 100× coverage from both PacBio and Illumina platforms to ensure complete and accurate genome assembly. Statistical methods were applied to analyze base distribution and quality fluctuations per sequencing cycle. Base quality, error rate, and distribution were assessed for each sample, enabling macro-level evaluation of library construction and sequencing quality.

The cleaned high-quality data were assembled using Unicycler v0.5.1 with PacBio long reads, and Pilon v3.12.2 was employed for sequence polishing. When terminal overlaps exceeding a defined length were detected in the assembled sequence, circularization was performed with trimming of one overlapping region. This process yielded complete chromosomal and plasmid sequences. Coding sequences (CDSs) were predicted using Glimmer v3.02, GeneMarkS v4.28, and Prodigal v2.5.3. Chromosomal genes were annotated with Prodigal, whereas plasmid genes were predicted using GeneMarkS v4.28. The tRNA genes were identified using tRNAscan-SE v2.0, which provided information on nucleotide sequences, anticodons, and secondary structures. The rRNA genes were predicted using Barrnap v3, yielding details on type, genomic location, and sequence for all rRNAs in each genome.

Species identification of the isolated CHHM-1 was conducted based on its genome sequence, utilizing the Genome Taxonomy Database (GTDB, https://gtdb.ecogenomic.org/tools/fastani, accessed on 9 July 2025). The Average Nucleotide Identity (ANI) between the isolate CHHM-1 genome and those of its nine closest type-strain relatives retrieved from the GTDB was calculated using the GTDB-Tk toolkit v2.4.1. Species assignment was established using the widely accepted ANI threshold of ≥95% for species demarcation.

### 2.6. Assessment of Genomic Similarity and Collinearity

For comparative genomic analysis, whole-genome sequences of five representative *P. koreensis* strains were downloaded from the NCBI database (https://www.ncbi.nlm.nih.gov/, accessed on 22 August 2025): BM06 (GenBank accession: CP155621.1; source: Nanjing, China), BS3658 (LT629687.1; DOE Joint Genome Institute), P19E3 (CP027477.1; Boppelsen, Switzerland), CRS05-R5 (CP015852.1; Hangzhou, China), and FP1691 (CP111114.1; Nanning, China).

ANI analysis was performed using JSpecies online tool (https://jspecies.ribohost.com/jspeciesws/#analyse, accessed on 22 August 2025). Based on the resulting ANI value matrix, a heatmap was generated with the ChiPlot online tool (https://www.chiplot.online/, accessed on 22 August 2025) to visually represent genomic similarities among strains.

Genomic collinearity analysis was conducted using Mauve v2.4.0. The results were visualized through locally collinear blocks (LCBs), where colors and arrangements intuitively display structural variations among strains. These visualizations reveal conserved regions, gene rearrangements, and insertion/deletion events.

### 2.7. Gene Annotation and Functional Prediction

Gene prediction was performed by aligning the sequenced genomes against multiple databases, including the Non-Redundant Protein Sequence Database (NR, https://www.ncbi.nlm.nih.gov/refseq/about/nonredundantproteins/, accessed on 4 July 2025), Swiss-Prot (https://www.uniprot.org/, accessed on 4 July 2025), Pfam (http://pfam.xfam.org/, accessed on 4 July 2025), Clusters of Orthologous Groups of Proteins (COG, https://www.ncbi.nlm.nih.gov/research/cog/, accessed on 4 July 2025), Gene Ontology (GO, http://geneontology.org/, accessed on 4 July 2025), and the Kyoto Encyclopedia of Genes and Genomes (KEGG, https://www.genome.jp/kegg/, accessed on 4 July 2025), using Diamond with an E-value threshold of ≤1 × 10^−5^.

Antibiotic resistance genes were identified by aligning sequences against the Comprehensive Antibiotic Resistance Database (CARD, https://card.mcmaster.ca/, accessed on 4 July 2025) using Diamond, with minimum coverage and identity set at 80% and an E-value ≤ 1 × 10^−5^.

### 2.8. Determination of Plant Growth-Promoting Traits In Vitro and in A. arguta

Cellulase production: the isolate CHHM-1 was inoculated into cellulose Congo red medium to determine cellulolytic activity [14], with cellulose as the sole carbon source. After 7 days of incubation at 25 °C, the formation of a clear zone indicated positive cellulase production. The test was repeated twice with three replicates.

Siderophore production: the isolate CHHM-1 was inoculated onto chromium azurophil S (CAS) agar to determine the secretion of iron carriers [14]. Plates were incubated at 25 °C for 7 days, with color changes around colonies indicating siderophore secretion.

Phosphate solubilization assay: the isolate CHHM-1 was inoculated into the National Botanical Research Institute’s Phosphate (NBRIP) medium containing tricalcium phosphate (TCP) as the sole phosphorus source to assess phosphate solubilizing ability [14]. After incubation at 25 °C for 7 days, the formation of a clear zone indicated positive phosphate solubilization.

Nitrogen fixation assay: the nitrogen-fixing ability was assessed using Ashby solid medium [15,16]. Firstly, the isolate CHHM-1 was passaged twice in Ashby solid medium and then inoculated onto fresh plates. After 7 days of incubation at 25 °C, bacterial growth was observed; growth on this nitrogen-free medium indicated positive nitrogen fixation capability.

Protease production: the isolate CHHM-1 was inoculated into skimmed milk solid medium to determine protease production [14]. After incubation at 25 °C for 7 days, protease production was indicated by the formation of a transparent hydrolysis halo around the colonies.

Ammonia production assay: the isolate CHHM-1 was inoculated into peptone ammoniated medium and incubated at 25 °C for 7 days [17]. The uninoculated peptone ammonia medium was used as a control. After incubation, 3–5 drops of Nessler’s reagent were added to the plate. If a yellow or brownish-red precipitate was produced, it indicated that the strain could produce ammonia. No color change was observed in the uninoculated control.

To determine the ability of indole-3-acetic acid (IAA) production, the isolate CHHM-1 was inoculated into LB medium containing 5 mmol/L of L-tryptophan and incubated at 25 °C for 7 days. After incubation, 1 mL of the culture supernatant was mixed with 1.5 mL of Salkowski’s reagent, and the mixture was left at room temperature for 30 min [14]. The appearance of a pink color in the test sample was recorded as a positive for IAA production. Assay validation was performed using both a sterile medium negative control, which remained colorless, and an IAA-supplemented positive control, which developed the expected pink hue.

A plant growth promotion assay was conducted using *A. arguta* plantlets. The isolate CHHM-1 was grown in LB liquid medium at 28 °C, reaching the late log phase following 12 h of growth. Bacterial cells were harvested by centrifugation at 966× *g* using an Allegra 64R high-speed refrigerated centrifuge equipped with an F0850 fixed-angle rotor and washed twice with sterile water. The resulting pellet was resuspended in sterile water to prepare the inoculum. The roots of three-month-old *A. arguta* plantlets were immersed in the bacterial suspension for 2 min. Control plantlets of equivalent growth status were treated with sterile water using the same method. The experiment included thirty biological replicates. After 7 days of treatment, plant fresh weight, leaf number, and plant height were measured.

### 2.9. Determination of Protective and Therapeutic Effects of the Isolate CHHM-1 Against P. syringae *pv.* actinidiae

To evaluate the biocontrol potential of the isolate CHHM-1 against bacterial canker in *A. arguta*, pathogenicity assays were conducted under both protective and therapeutic inoculation regimens using *P. syringae* pv. *actinidiae*. Two in vitro bioassay methods were employed according to established protocols: (1) inoculation of detached *A. arguta* shoots with bacterial suspensions at 10^8^ CFU/mL following Zhao et al. [18], and (2) immersion of *A. arguta* leaf discs in bacterial suspensions at 10^8^ CFU/mL following Tian et al. [19].

For the detached shoot assay, healthy woody shoots of *A. arguta* were cut into 10 cm segments, surface-sterilized with 0.6% sodium hypochlorite for 10 min and rinsed three times with sterile water. After air-drying, a uniform wound was created on each segment using a sterile scalpel.

The following treatments were applied: (1) water control: 10 μL of sterile water applied to the wound site; (2) CHHM-1 control: 10 μL of CHHM-1 suspension (10^8^ CFU/mL) applied to the wound site; (3) *P. syringae* pv. *actinidiae* + water: 10 μL of *P. syringae* pv. *actinidiae* suspension (10^8^ CFU/mL) applied to the wound site, followed by 10 μL of sterile water 24 h later; (4) *P. syringae* pv. *actinidiae* + CHHM-1 (therapeutic): 10 μL of *P. syringae* pv. *actinidiae* suspension (10^8^ CFU/mL) applied to the wound site, followed by 10 μL of CHHM-1 suspension (10^8^ CFU/mL) 24 h later; (5) water + *P. syringae* pv. *actinidiae*: 10 μL of sterile water applied to the wound site, followed by 10 μL of *P. syringae* pv. *actinidiae* suspension (10^8^ CFU/mL) 24 h later; (6) CHHM-1 + *P. syringae* pv. *actinidiae* (preventive): 10 μL of CHHM-1 suspension (10^8^ CFU/mL) applied to the wound site, followed by 10 μL of *P. syringae* pv. *actinidiae* suspension (10^8^ CFU/mL) 24 h later. Lesion length was measured 7 days post-inoculation.

For the leaf disc assay, leaves were surface-sterilized with 0.6% sodium hypochlorite, rinsed with sterile water, and cut into 10 mm diameter discs using a sterile punch. The following treatments were applied:

(1) Water control: discs immersed in sterile water for 20 s before transfer to Petri dishes lined with moist sterile filter paper; (2) CHHM-1 control: discs immersed in CHHM-1 suspension (10^8^ CFU/mL) for 20 s before transfer to Petri dishes lined with moist sterile filter paper; (3) *P. syringae* pv. *actinidiae* + water: discs immersed in *P. syringae* pv. *actinidiae* suspension (10^8^ CFU/mL) for 20 s. After 24 h, discs were re-immersed in sterile water for 20 s and returned to Petri dishes; (4) *P. syringae* pv. *actinidiae* + CHHM-1 (therapeutic): discs immersed in *P. syringae* pv. *actinidiae* suspension (10^8^ CFU/mL) for 20 s. After 24 h, discs were treated with CHHM-1 suspension (10^8^ CFU/mL) by immersion for 20 s and returned to Petri dishes; (5) water + *P. syringae* pv. *actinidiae*: discs immersed in sterile water for 20 s. After 24 h, discs were immersed in *P. syringae* pv. *actinidiae* suspension (10^8^ CFU/mL) for 20 s and returned to Petri dishes; (6) CHHM-1 + *P. syringae* pv. *actinidiae* (preventive): discs immersed in CHHM-1 suspension (10^8^ CFU/mL) for 20 s. After 24 h, discs were immersed in *P. syringae* pv. *actinidiae* suspension (10^8^ CFU/mL) for 20 s and returned to Petri dishes. Disease severity was assessed 5 days post-inoculation by measuring lesion area.

### 2.10. Preparation of Bacterial Culture Filtrate (BF)

The isolate CHHM-1 was inoculated into LB medium on a shaker (120 rpm) at 28 °C for 5 d. The bacterial suspension was then centrifuged at 3000 rpm (966× *g*; Allegra 64R centrifuge with F0850 rotor, Beckman Coulter, Inc., Shanghai, China) at 4 °C for 20 min, and the supernatant was sequentially filtered through 0.22 μm membranes to obtain a cell-free sterile filtrate (BF) [12].

### 2.11. Assessment of Antibacterial Activity and Morphological Effects of BF

The antibacterial activity of BF was evaluated by monitoring the growth curve of *P. syringae* pv. *actinidiae* following treatment [12]. *P. syringae* pv. *actinidiae* was pre-cultured in LB medium at 28 °C for 12 h to an OD_600_ of 0.25 (~6.5 × 10^7^ CFU/mL). This culture was then inoculated into fresh LB medium at a 1:6 (*v*:*v*) ratio. BF was added to achieve final concentrations of 25% and 50% (*v*/*v*). In the control group, *P. syringae* pv. *actinidiae* was inoculated into LB containing the same amount of fermentation medium. All cultures were incubated at 28 °C with shaking (120 rpm) for 48 h. The optical density at 600 nm was recorded every 4 h. Growth curves were plotted with time as the horizontal coordinate and OD_600_ as the vertical coordinate, using the 0 h measurement as the baseline. The experiment included three replicates per treatment and was conducted twice.

For SEM observation, *P. syringae* pv. *actinidiae* cultures from the control and 50% BF treatment groups were collected after 24 h of incubation and processed according to the procedures described in Section 2.12 [12].

### 2.12. Scanning Electron Microscopy Sample Preparation

SEM was used to observe bacterial cell morphology and ultrastructural changes [12].

Bacterial cells were harvested by centrifugation, washed with 0.1 M PBS (pH 7.0), and fixed in 4 °C pre-cooled fixative overnight at 4 °C. After fixation, samples were rinsed with phosphate buffer (pH 7.0) and post-fixed in 1% osmium tetroxide for 1–2 h.

Dehydration was carried out through a graded ethanol series (30%, 50%, 70%, 80%, 90%, 95%, 100%), followed by treatment with ethanol/isoamyl acetate (1:1) and pure isoamyl acetate. Samples were critical-point dried, sputter-coated with gold, and examined by SEM.

### 2.13. Data Processing and Analysis

Statistical analysis was performed using SPSS v.20.0 with one-way ANOVA and LSD post hoc tests. Graphs were generated using Excel 2021 and Origin 2018, while figure layouts were prepared with Adobe Illustrator CS6. All experiments included at least three independent biological replicates unless otherwise specified.

## 3. Results and Analysis

### 3.1. Antagonistic Activity of Rhizosphere Bacteria Against P. syringae *pv.* actinidiae

**Figure 1:**
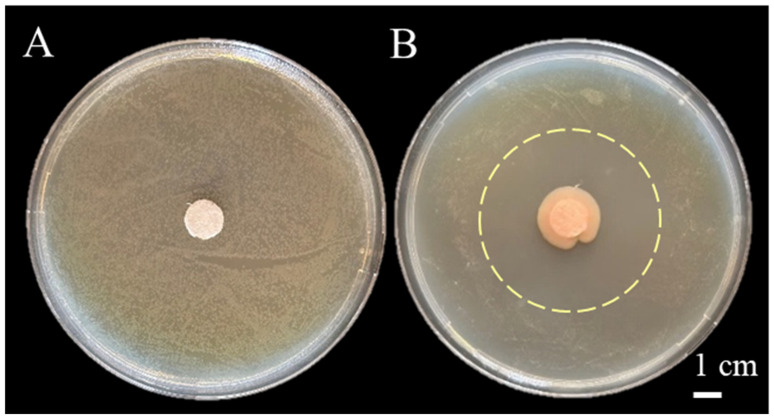
Inhibitory effect of *Pseudomonas koreensis* CHHM-1 on *Pseudomonas syringae* pv. *actinidiae.* A *P. syringae* pv. *actinidiae* suspension (~7 × 10^7^ CFU/mL, 10^4^ dilution) was diluted 10^4^-fold and spread on NA medium. (**A**) Control: A sterile filter disc loaded with 10 μL sterile water shows no inhibition zone against the lawn of *P. syringae* pv. *actinidiae*. (**B**) CHHM-1 treatment: A sterile filter disc loaded with 10 μL of a CHHM-1 culture. A distinct inhibition zone (mean diameter: 4.36 cm), indicated by the pale-yellow dashed circle, demonstrates the antagonistic activity of the isolate CHHM-1. The large central colony corresponds to the *P. koreensis* CHHM-1 colony originating from the inoculum on the disc.

A total of 106 bacterial strains were isolated from rhizosphere soil on NA medium, among which only four exhibited inhibitory activity against *P. syringae* pv. *actinidiae*. The isolate CHHM-1, which showed the strongest antagonistic effect, was selected for further investigation. Antagonistic activity was assessed in NA medium using a dual-culture plate assay. The assay revealed that the isolate CHHM-1 significantly suppressed the growth of *P. syringae* pv. *actinidiae*, as evidenced by a distinct inhibition zone observed within 7 days of co-cultivation (Figure 1A,B). This inhibition zone had an average diameter of 4.36 cm (Figure 1B), confirming the strain’s potent antagonistic capability.

To evaluate the biocontrol potential against the *P. syringae* pv. *actinidiae* strains isolated from kiwifruit canker, the same dual-culture plate assay protocol was applied. The results demonstrated that the isolate CHHM-1 exhibited significant antagonistic activity against *P. syringae* pv. *actinidiae* from kiwifruit canker (Supplementary Figure S1).

### 3.2. Identification of Rhizobacteria

**Figure 2:**
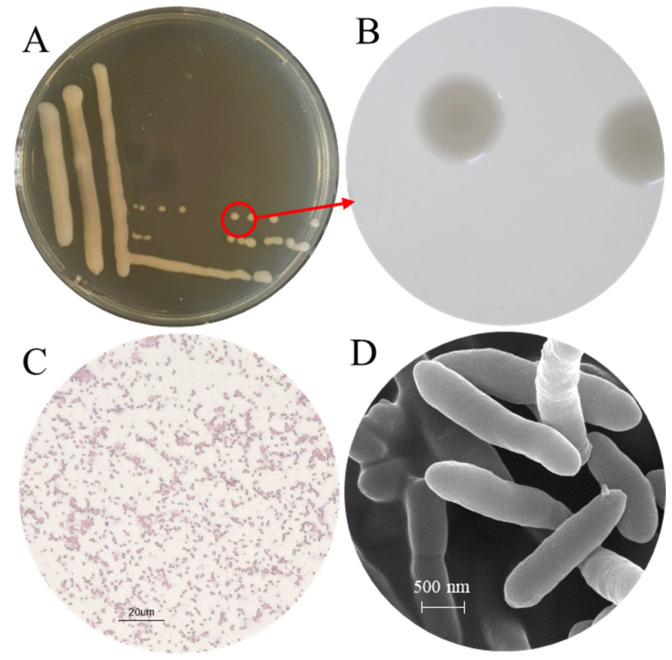
Colony and cellular morphology of *P. koreensis* CHHM-1. (**A**) Colony morphology on nutrient agar (NA) after 48 h incubation at 28 °C. (**B**) Close-up view of a single colony (outlined in red in panel (**A**)), revealing detailed morphology. (**C**) Micrograph of Gram-stained cells, showing the Gram-negative reaction. (**D**) Scanning electron micrograph (SEM) showing rod-shaped cells with tapered ends, typical of pseudomonad morphology.

The morphological characteristics of the isolate CHHM-1 were analyzed. After growth on NA medium at 28 °C for 48 h, the colonies were round, moist, convex, and glossy, with smooth margins and pale-yellow pigmentation (Figure 2A,B). They measured 0.5–2.0 mm in diameter, emitted a distinct earthy odor, and displayed rapid growth rates.

The isolate CHHM-1 was identified as a Gram-negative bacterium (Figure 2C). SEM further showed that the planktonic CHHM-1 cells were long rods with tapered ends, measuring 1.7–2.1 μm in length and 0.45–0.55 μm in width, a morphology consistent with that of typical pseudomonads (Figure 2D).

The physiological and biochemical characteristics of the isolate CHHM-1 were determined as shown in Table 1.

**Figure 3:**
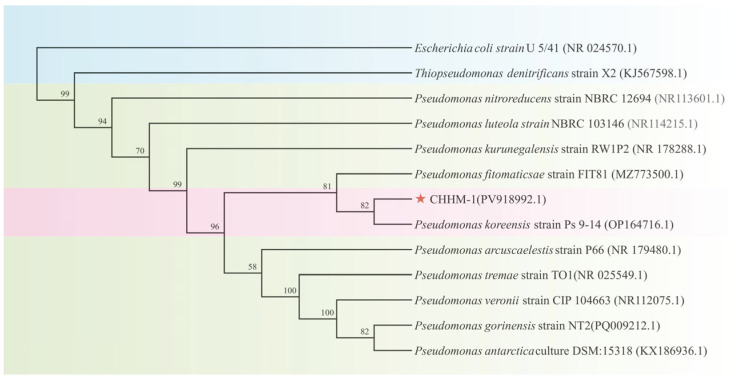
Phylogenetic tree of the isolate CHHM-1 based on 16S rRNA gene sequences. The tree was constructed using the maximum-likelihood method, showing the evolutionary relationships among the isolate CHHM-1 (indicated by a red star) and type strains of *Pseudomonas*. To clarify taxonomic groupings, distinct background colors are applied: a pink background highlights the clade comprising the isolate CHHM-1 and *P. koreensis* strain RZ98, confirming their conspecific status; a light green background denotes other species within the genus Pseudomonas; and a blue background identifies the outgroup species. Bootstrap values (based on 1000 replicates) greater than 50% are shown at branch nodes. The isolate CHHM-1 clusters with *P. koreensis* strain RZ98 with 100% bootstrap support, confirming its taxonomic placement. NCBI accession numbers are provided in parentheses. *Escherichia coli* strain U 5/41 (NR024570) was used as the outgroup.

The identification of the isolate CHHM-1 was confirmed through molecular analysis. A 1448 bp fragment of the 16S rRNA gene was amplified and sequenced (GenBank accession: PV918992.1). The sequence was compared with the GenBank database using NCBI (https://blast.ncbi.nlm.nih.gov/Blast.cgi, accessed on 10 May 2025) BLAST, revealing 100% similarity to *P. koreensis* strain RZ98 (GenBank accession: OP164716.1). Phylogenetic analysis using the maximum likelihood method revealed that the isolate CHHM-1 clusters within the same clade as the *P. koreensis* strain RZ98 with a high bootstrap support of 100% (Figure 3), confirming their close phylogenetic relationship.

The isolate CHHM-1 was classified within the genus *Pseudomonas* according to the Genome Taxonomy Database (GTDB). Whole-genome sequencing revealed that the isolate CHHM-1 possesses a single circular chromosome of 6,181,985 bp, and subsequent ANI analysis showed 98.12% identity with *P. koreensis*, exceeding the 95% species delineation threshold (Table 2). This molecular evidence, together with the morphological characteristics, definitively identifies the isolate CHHM-1 as a strain of *P. koreensis*.

### 3.3. Whole-Genome Analysis of P. koreensis CHHM-1

**Figure 4:**
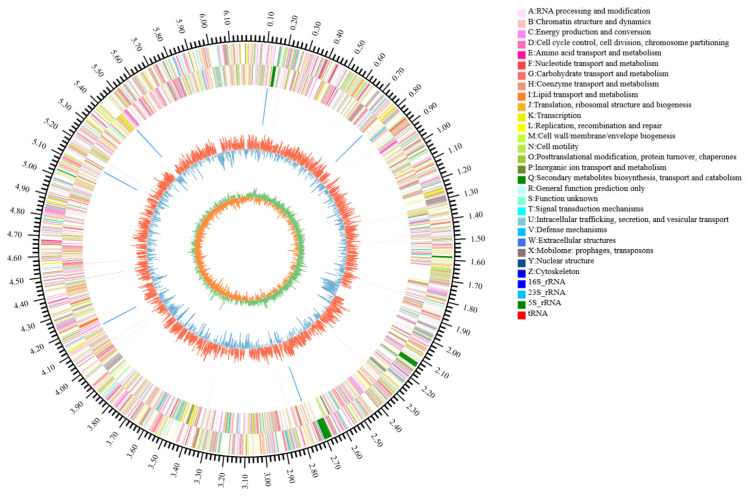
Circular genome visualization of *P. koreensis* CHHM-1. The concentric circles represent (from outer to inner): (1) genome size scale, (2) forward-strand genes (3) reverse-strand genes, (4) rRNA/tRNA distribution (5) GC content variation (red outward peaks indicate regions exceeding the 60.36% average GC content; blue inward troughs denote regions below average), and (6) GC skew profile.

The complete genome of the isolate CHHM-1 comprises a single circular chromosome of 6,181,985 bp (BioSample accession: CP196684.1) and has a GC content of 60.36%. A total of 47,987 sequencing reads were obtained, with an average read length of 10,367.76 bp and an N50 of 10,299 bp; the longest read was 29,007 bp. Genome annotation predicted 5448 protein-coding sequences with a total length of 5,421,669 bp, representing 87.70% of the genome. Additionally, 73 tRNA and 19 rRNA genes were identified, including 6 copies of 16S rRNA, 6 copies of 23S rRNA, and 7 copies of 5S rRNA. A Circos plot illustrating the genome characteristics is shown in Figure 4.

### 3.4. Comparative Genomic Analysis

To gain deeper insights into the genomic diversity of *P. koreensis*, a comparative analysis of six *P. koreensis* strains from diverse geographical origins was performed. The results (Table 3) indicated similar genome sizes among the six strains, ranging from 5.9 to 7.5 Mb, and highly consistent GC contents (approximately 59.0–60.5%), reflecting conserved basic genomic features.

**Figure 5:**
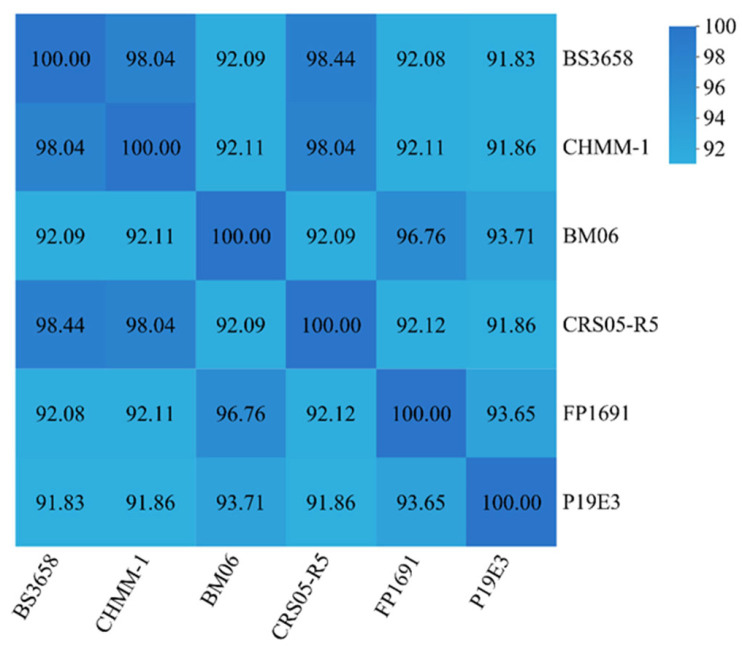
ANI among *P. koreensis* strains. Heatmap showing pairwise ANI values between the isolate CHHM-1 and five other *P. koreensis* strains. The isolate CHHM-1 shows high genomic similarity (98.04%) with strains BS3658 and CRS05-R5, exceeding the 95% species threshold. In contrast, ANI values with strains BM06, P19E3, and FP1691 are significantly lower (91.83–92.11%), indicating substantial intraspecific divergence.

ANI analysis was performed using JSpecies online tool (https://jspecies.ribohost.com/jspeciesws/#analyse, accessed on 22 August 2025) to compare the isolate CHHM-1 with other *Pseudomonas* strains (Figure 5). The isolate CHHM-1 showed 98.04% ANI with both *P. koreensis* BS3658 and *P. koreensis* CRS05-R5, confirming high similarity at the species level. In contrast, ANI values with *P. koreensis* BM06, P19E3, and FP1691 were lower (91.86–92.11%), falling below the 95% species threshold and indicating significant genetic divergence. The ANI heatmap clearly illustrated these hierarchical similarity relationships, providing insights into the intraspecific genetic structure of *P. koreensis*.

**Figure 6:**
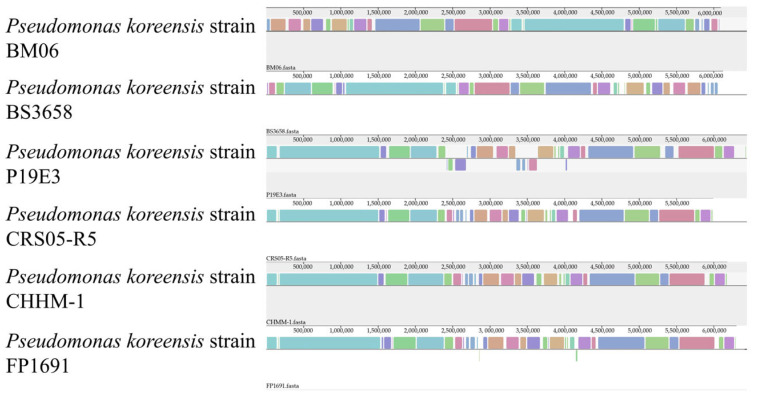
Genomic collinearity analysis of six *P. koreensis* strains. The plot shows genomic alignments between the isolate CHHM-1 and five other *P. koreensis* strains. Locally collinear blocks (LCBs) are represented by colored segments. The isolate CHHM-1 and CRS05-R5 show high collinearity, indicating strong genomic conservation. Gaps in the alignment represent potential deletions or absent regions in the isolate CHHM-1 compared to other strains.

Collinearity analysis was performed between the isolate CHHM-1 and five other *P. koreensis* strains. The results indicated a high degree of collinearity between CHHM-1 and CRS05-R5, as most locally collinear blocks (LCBs) were highly conserved between these two strains (Figure 6). These conserved regions are likely to contain essential core genes, such as those for central metabolism or cellular structure. However, genomic rearrangements including deletions, inversions, and translocations were also observed. Notably, gaps in the isolate CHHM-1 genome indicate the absence of specific genomic regions present in other strains.

### 3.5. Basic Genomic Annotation of P. koreensis CHHM-1

**Figure 7:**
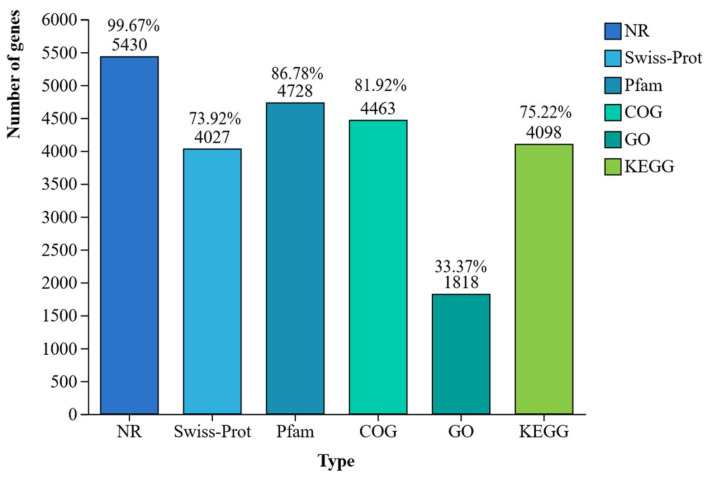
Functional annotation of protein-coding genes in *P. koreensis* CHHM-1. This bar chart showing the proportion and number of successfully annotated genes out of the total 5448 protein-coding genes in the isolate CHHM-1 genome across six major databases: NR (5430; 99.67%), Swiss-Prot (4027; 73.92%), Pfam (4728; 86.78%), COG (4463; 81.92%), GO (1818; 33.37%), and KEGG (4098; 75.22%).

The isolate CHHM-1 possesses 5448 protein-coding genes, with 5430 (99.67%), 4027 (73.92%), 4728 (86.78%), 4463 (81.92%), 1818 (33.37%), and 4098 (75.22%) successfully annotated in the NR, Swiss-Prot, Pfam, COG, GO, and KEGG databases, respectively (Figure 7).

**Figure 8:**
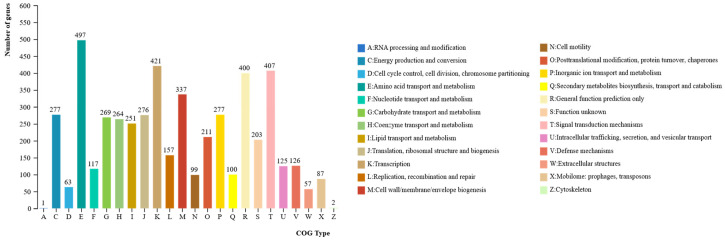
*P. koreensis* CHHM-1 genome gene function COG database annotation. The bar chart shows the distribution of 4463 annotated genes across 24 COG functional categories. The most abundant functional categories include amino acid transport and metabolism (497 genes), Transcription (421 genes), replication, recombination and repair (407 genes), General function prediction (400 genes), and Cell wall/membrane/envelope biogenesis (337 genes).

Functional annotation of *P. koreensis* CHHM-1 protein-coding genes in the COG database revealed 4463 annotated genes (81.92% of total genes), classified into 24 functional categories (Figure 8). The most abundant categories were amino acid transport and metabolism (497 genes), Transcription (421 genes), Replication, recombination and repair (407 genes), General function prediction only (400 genes), Cell wall/membrane/envelope biogenesis (337 genes), Cell cycle control, cell division, chromosome partitioning (277 genes), Inorganic ion transport and metabolism (277 genes), Translation, ribosomal structure and biogenesis (276 genes), Carbohydrate transport and metabolism (269 genes), Coenzyme transport and metabolism (264 genes), Lipid transport and metabolism (251 genes).

**Figure 9:**
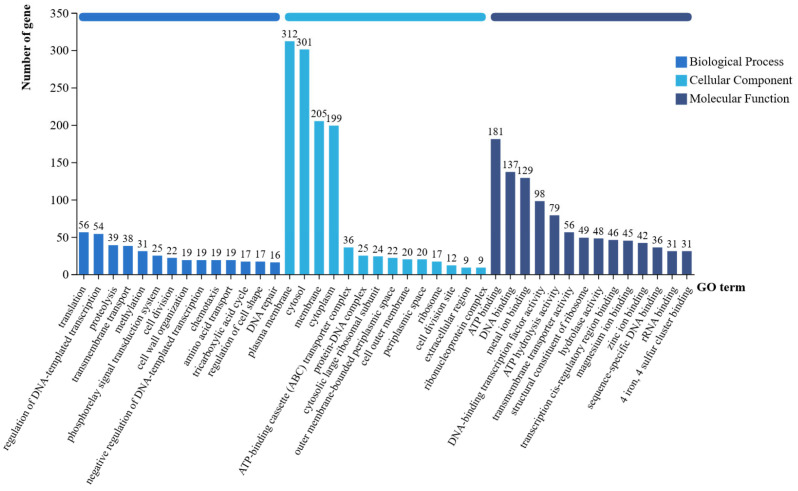
Annotation of the GO database on gene function of the *P. koreensis* CHHM-1 genome. The x-axis displays the three primary GO categories—Biological Process (BP), Cellular Component (CC), and Molecular Function (MF)—along with their respective level 2 subcategories, while the y-axis represents the number of annotated genes.

Comparative analysis of the *P. koreensis* CHHM-1 genome against the GO database annotated 1818 functional genes (Figure 9), with 1167, 1220, and 1505 genes assigned to Biological Process, Cellular Component, and Molecular Function categories, respectively.

The annotated genes were primarily associated with cellular membrane components, energy metabolism, and transport activity. Additionally, genes involved in siderophore synthesis, biofilm formation, and secretion systems were identified, along with genes related to nitrogen fixation, phosphate solubilization, and phytohormone biosynthesis, consistent with its plant growth-promoting rhizobacterium characteristics.

**Figure 10:**
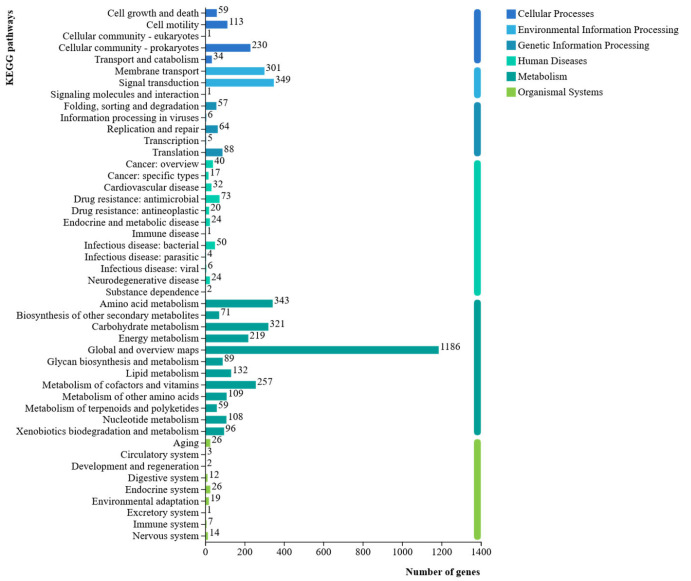
*P. koreensis* CHHM-1 genome gene function KEGG database annotation. Bar chart showing the distribution of annotated genes across the six major KEGG functional categories. A total of 4098 protein-coding genes (75.22% of the total genes) were assigned to 46 metabolic pathways, high-lighting the strain’s genomic potential for diverse metabolic functions and environmental adaptation.

KEGG database analysis annotated 4098 protein-coding genes from the isolate CHHM-1 genome (representing 75.22% of total genes) across 46 metabolic pathways (Figure 10). These genes were enriched in six major functional categories: Cellular Processes, Environmental Information Processing, Metabolism, Genetic Information Processing, Organismal Systems, and Human Diseases. The predominant pathway annotations included Global and overview maps (1186 genes), Signal transduction (349 genes), Amino acid metabolism (343 genes), Carbohydrate metabolism (321 genes), and Membrane transport (301 genes), demonstrating significant genomic potential for metabolic versatility and environmental adaptation.

### 3.6. Prediction of Antibiotic Resistance Genes

Analysis using the Comprehensive Antibiotic Resistance Database (CARD) predicted 11 antibiotic resistance genes in the isolate CHHM-1 genome, with a minimum sequence identity of 80% (Table 4). The strain exhibited broad-spectrum resistance potential, with fluoroquinolone resistance genes present across all predicted categories, representing a core resistance trait. High-frequency occurrences of phenol and diaminopyrimidine resistance genes were also observed. These multidrug resistance characteristics may reflect long-term antibiotic selection pressures in agricultural environments (Supplementary Table S1).

The presence of these predicted resistance genes suggests that the isolate CHHM-1 may possess multidrug resistance capabilities, which could theoretically confer a survival advantage in orchard environments with routine pesticide application. However, these genomic predictions require experimental validation to confirm actual phenotypic resistance.

### 3.7. Plant Growth-Promoting Traits of the Isolate CHHM-1

In vitro assays demonstrated that the isolate CHHM-1 exhibits multiple plant growth-promoting properties, including nitrogen fixation, ammonia production, phosphate solubilization, and secretion of siderophores, indole-3-acetic acid (IAA), and proteases, though cellulase activity was not detected.

**Figure 11:**
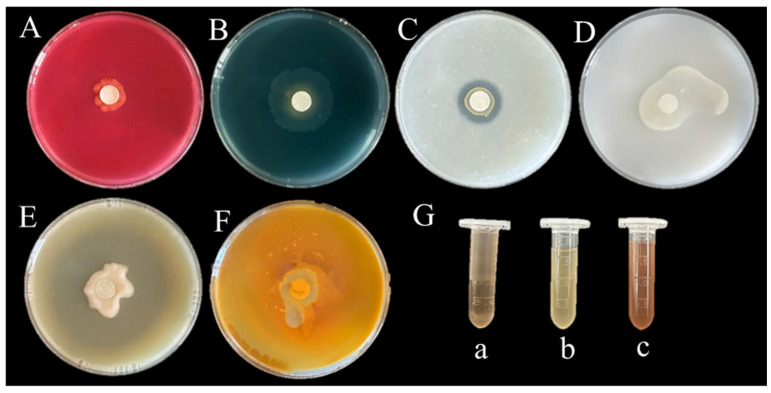
Functional validation of plant growth-promoting traits in *P. koreensis* CHHM-1. (**A**) Cellulase activity; (**B**) siderophore secretion; (**C**) phosphate solubilization; (**D**) nitrogen fixation; (**E**) protease activity; (**F**) ammonia production; (**G**) IAA production assay using Salkowski reagent. (**a**) Positive control (IAA standard). (**b**) negative control (sterile medium). (**c**) CHHM-1 culture supernatant with positive pink coloration.

Functional characterization assays demonstrated that the isolate CHHM-1 exhibited multiple plant growth-promoting traits (Figure 11): (A) no halo formation on cellulose Congo red medium, indicating absence of cellulase production; (B) yellow halos on CAS agar, confirming siderophore synthesis; (C) clearance zones on NBRIP solid medium, demonstrating phosphate solubilization capacity; (D) growth on nitrogen-free Ashby medium, verifying nitrogen fixation ability; (E) transparent halos on skim milk agar, evidencing protease activity; (F) yellow-brown precipitation with Nessler’s reagent, indicating ammonia production; and (G) pink coloration with Salkowski reagent in culture supernatant, confirming IAA biosynthesis. These in vitro results establish the isolate CHHM-1 as a multifunctional biofertilizer capable of promoting plant growth through IAA secretion, phosphate solubilization, and nitrogen fixation, thereby enhancing root development and seedling vigor.

Inoculation with the isolate CHHM-1 significantly promoted the growth of *A. arguta* compared to the non-inoculated control. Plant fresh weight increased by 32.1–68.3% (*p* < 0.05), and leaf number increased by 45.3–75.1% (*p* < 0.05). In contrast, plant height showed no statistically significant increase (Supplementary Figure S2).

### 3.8. The Determination of the Preventive and Therapeutic Effects of the Isolate CHHM-1 on P. syringae *pv.* actinidiae

The efficacy of the isolate CHHM-1 in controlling bacterial canker was evaluated using detached shoot and leaf disc assays of *A. arguta*. The lesion area was assessed 5 days post-inoculation in leaf discs, and lesion length was measured 7 days post-inoculation in shoots.

**Figure 12:**
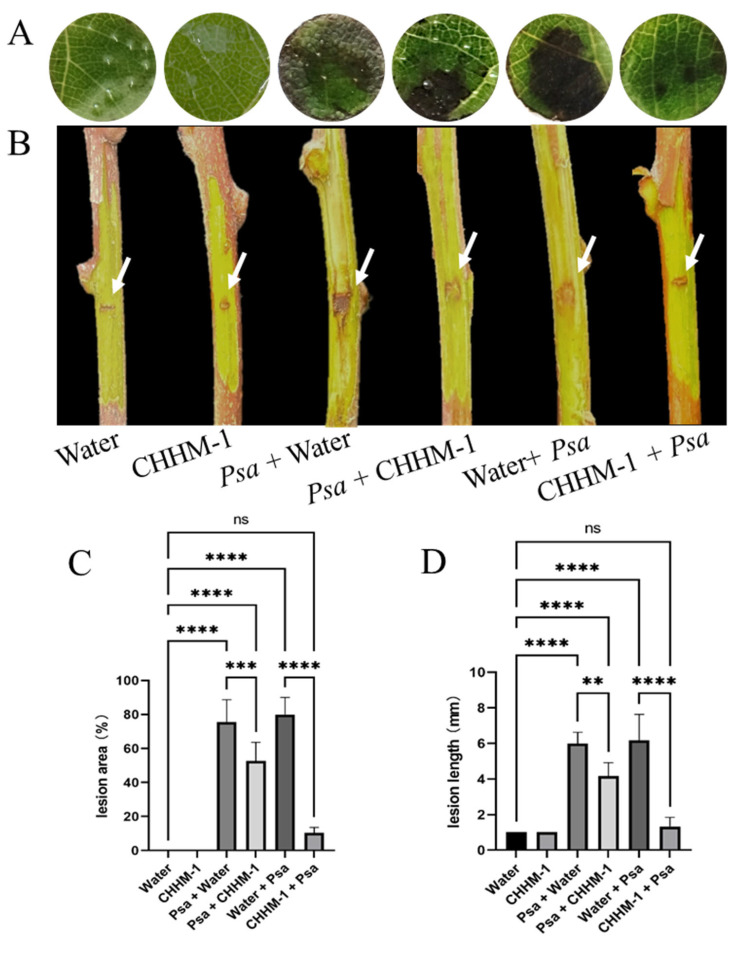
The protective and therapeutic efficacy of the isolate CHHM-1 in vitro. In the figure, Psa refers to *P*. *syringae* pv. *actinidiae*. (**A**) Representative images of leaf discs showing lesion development 5 days post-inoculation. (**B**) Representative images of detached shoots showing lesion progression 7 days post-inoculation. White arrows indicate wound sites created by sterile scalpel incision; the minimal lesions observed in the water control and CHHM-1 control groups were attributed to the mechanical wounding procedure rather than pathogenic infection. (**C**) Quantitative analysis of lesion area on leaf discs. (**D**) Quantitative analysis of lesion length on detached shoots. Data are presented as mean ± SD. Significance was determined by one-way ANOVA with Tukey’s test (ns: *p* > 0.05, **: *p* < 0.01, ***: *p* < 0.001, ****: *p* < 0.0001).

In the leaf disc assay (Figure 12A), no lesions were observed in either the water control or CHHM-1 control groups (recorded as 0% lesion area), whereas the lesion areas for the pathogen-inoculated groups *P. syringae* pv. *actinidiae* + water and water + *P. syringae* pv. *actinidiae* were 75.5% and 80%, respectively. In the therapeutic group (*P. syringae* pv. *actinidiae* + CHHM-1), the lesion area was 52.7%, showing a partial inhibitory effect. By contrast, in the preventive group (CHHM-1 + *P. syringae* pv. *actinidiae*), the lesion area was markedly reduced to 10%, indicating significant suppression of *P. syringae* pv. *actinidiae* development (Figure 12C).

In the detached shoot assay (Figure 12B), the water control and CHHM-1 control were 1 mm, while those for *P. syringae* pv. *actinidiae* + water and water + *P. syringae* pv. *actinidiae* were 6 mm and 6.2 mm, respectively. In the therapeutic treatment group (*P. syringae* pv. *actinidiae* + CHHM-1), the lesion length was 4.2 mm, indicating a moderate inhibitory effect. In the preventive treatment group (CHHM-1 + *P. syringae* pv. *actinidiae*), the lesion length was significantly reduced to 1.3 mm, demonstrating substantial suppression of *P. syringae* pv. *actinidiae* progression (Figure 12D).

### 3.9. Growth Inhibition of P. syringae *pv.* actinidiae by BF

**Figure 13:**
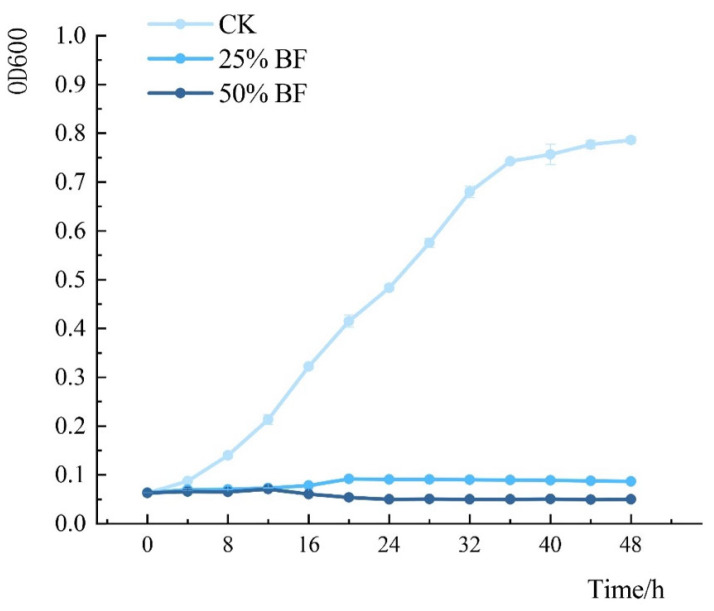
Growth dynamics of *P. syringae* pv. *actinidiae* after treatment with BF. Optical density at 600 nm (OD_600_) was measured over time for the untreated control (CK), 25% BF, and 50% BF treatment groups. Values represent means of three replicates; error bars indicate standard error (*n* = 3). The control group showed typical growth phases (lag, logarithmic, and stationary). In contrast, 25% BF severely inhibited growth, reducing the final optical density and delaying the logarithmic phase. The 50% BF treatment completely suppressed growth, with OD_600_ declining after 16 h, indicating a bactericidal effect.

The growth of *P. syringae* pv. *actinidiae* treated with different concentrations of BF was monitored by measuring optical density at 600 nm (Figure 13). BF treatment significantly suppressed *P. syringae* pv. *actinidiae* growth in a concentration-dependent manner, with higher BF concentrations leading to stronger inhibition. The untreated control (*P. syringae* pv. *actinidiae* cultured in sterile LB medium without BF) exhibited normal growth dynamics, whereas BF-treated groups showed delayed logarithmic phases and reduced final biomass.

In the control group, OD_600_ increased steadily, entering the logarithmic phase after 7 h and reaching the stationary phase at 33 h. In contrast, the 25% BF treatment showed delayed growth, with the logarithmic phase occurring between 16–20 h and a lower final OD_600_. The 50% BF treatment maintained consistently low OD_600_ values throughout the experiment, with a brief logarithmic phase (12–14 h) followed by a decline phase from 16 h onward. The dose-dependent reduction in bacterial density confirmed that BF strongly inhibits *P. syringae* pv. *actinidiae* growth in a concentration-dependent manner.

### 3.10. Morphological Changes in P. syringae pv. actinidiae Cells Induced by BF Treatment

**Figure 14:**
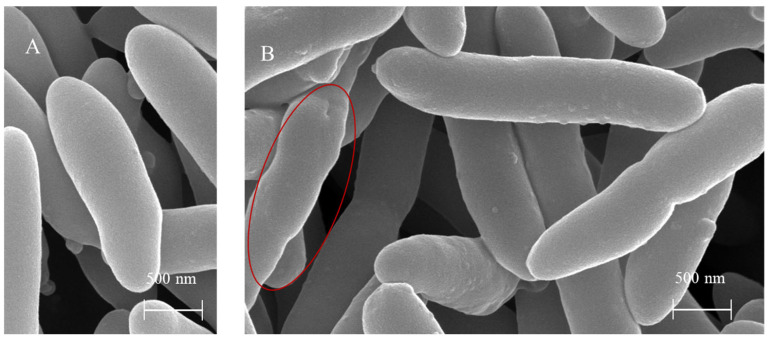
Scanning electron micrographs of *P. syringae* pv. *actinidiae* cells after 24 h incubation. (**A**) Untreated control cells in LB medium, showing intact rod-shaped morphology. (**B**) Cells treated with 50% BF in LB medium (28 °C, 120 rpm) displayed distinct non-lytic morphological changes, including severe deformation, extensive surface protrusions with scaly features, and cellular aggregation. The red oval highlights an area of pronounced distortion.

SEM revealed that BF treatment caused distinct non-lytic morphological changes in *P. syringae* pv. *actinidiae* cells (Figure 14). These included cell deformation, surface protrusion formation, and bacterial aggregation. These alterations contrast sharply with the intact rod-shaped morphology of untreated control cells, suggesting that BF may exert antimicrobial activity by disrupting bacterial membrane integrity.

## 4. Discussion

In the past, *P. syringae* pv. *actinidiae* has severely impacted global kiwifruit production, damaging not only susceptible cultivars like *A. chinensis var. deliciosa* (green kiwifruit) and *A. chinensis* var. *chinensis* (gold kiwifruit) [7] but also previously considered resistant species such as *A. arguta*, resulting in significant economic losses. Currently available treatments for bacterial canker caused by *P. syringae* pv. *actinidiae* are scarce, while the environmental toxicity of copper-based compounds and emerging antibiotic resistance issues necessitate the development of eco-friendly control strategies. In recent years, beneficial microorganisms have received increasing attention due to their significant advantages over traditional fungicides in terms of agricultural safety, economy, and sustainability, and disease management strategies based on biocontrol bacteria have shown broad application prospects [9].

Research on the biological control of bacterial canker, caused by *P. syringae* pv. *actinidiae*, has identified numerous antagonistic microorganisms with potential for disease management. These biocontrol resources are widely distributed across multiple genera, including *Acinetobacter*, *Bacillus*, *Chryseobacterium*, *Flavobacterium*, *Glutamicibacter*, *Lysinibacillus*, *Lysobacter*, and *Pseudomonas* [20]. Strains from *Pseudomonas* and *Bacillus* have attracted significant research interest due to their strong inhibitory activity and diverse modes of action. Current studies indicate that these biocontrol agents primarily suppress *P. syringae* pv. *actinidiae* through two distinct mechanisms: by secreting antimicrobial metabolites that disrupt the cellular structure of the pathogen [21,22,23,24,25,26], such as 2,4-diacetylphloroglucinol (DAPG) produced by *Pseudomonas bijieensis* XL17 [22] and fusaritricine produced by *Fusarium tricinctum* [25]; through direct contact-mediated inhibition, as exemplified by *Pantoea endophytica*, which employs a type VI secretion system (T6SS) to translocate toxic effector proteins into *P. syringae* pv. *actinidiae* cells, thereby inducing cell death [27]. Disruption of cell membrane integrity represents a well-documented antibacterial strategy. For instance, *Lonicera caerulea* polyphenols (LCPs) have been shown to alter the types of glycosidic bonds within exopolysaccharides (EPS), leading to a structurally compromised, rough, and porous EPS matrix. Concurrently, LCP inhibits the growth of *Streptococcus mutans*, reduces EPS and biofilm formation, and downregulates the expression of key biofilm-associated genes, collectively contributing to its effective bacterial suppression [28]. In this study, the isolate CHHM-1 exhibiting significant antagonistic activity against *P. syringae* pv. *actinidiae* was isolated from the rhizosphere soil of healthy *A. arguta* plants. Based on morphological characteristics and molecular sequencing analysis, the strain was identified as *P. koreensis*. Further cytological experiments confirmed that isolate CHHM-1 effectively inhibits *P. syringae* pv. *actinidiae* growth by secreting antibacterial substances. This finding not only provides a new microbial resource derived from the host plant rhizosphere for *P. syringae* pv. *actinidiae* biocontrol but also reinforces that secretion of antimicrobial compounds is an efficient and common mechanism employed by *Pseudomonas* species to suppress *P. syringae* pv. *actinidiae*. Consequently, the isolate CHHM-1 demonstrates promising potential for development as an environmentally friendly biopesticide.

While *P. koreensis* displays broad-spectrum antimicrobial properties, its antagonistic efficacy shows strain-specific variations. Regarding antibacterial activity, Kaur et al. previously isolated antimicrobial compounds from this species that were active against both Gram-positive and Gram-negative bacteria [29]. Multiple studies have confirmed the antagonistic activity of *P. koreensis* against fungal pathogens such as *Botrytis cinerea* [30], *Hymenoscyphus fraxineus* [31], and *Cephalosporium maydis* [32]. However, this study found no significant inhibitory effects against *Fusarium oxysporum*, *F. solani*, *Ilyonectria robusta*, or *F. graminearum* (Supplementary Figure S3), suggesting strain- or subspecies-dependent variations in antifungal specificity. The strain-specific variations in antimicrobial activity observed in *P. koreensis* likely originate from natural genetic diversity within the species, particularly in biosynthetic gene clusters responsible for antimicrobial compound production. These genomic differences may be further modulated by variations in regulatory networks that control metabolite expression and secretion [33]. Additionally, adaptive evolution in distinct ecological niches could select for specialized antimicrobial profiles, explaining the differential efficacy against specific fungal pathogens observed among strains.

Previous studies have shown that *P. koreensis* has been widely reported to exhibit multiple agricultural functions, including successful root colonization [30], mitigation of drought-induced physiological stress in plants [34,35,36], reduction of agrochemical and heavy metal toxicity [37,38], and plant growth promotion through nitrogen fixation, organic nitrogen ammonification, and phytohormone (e.g., IAA) biosynthesis [10]. This study further validated these growth-promoting mechanisms in the isolate CHHM-1, confirming its capabilities for nitrogen fixation, ammonia production, phosphate solubilization, and secretion of indole-3-acetic acid (IAA), siderophores, and proteases. Genomic analysis additionally identified multiple agriculturally relevant antibiotic resistance genes in the isolate CHHM-1. These traits align with previous findings on *P. koreensis* [10] while providing a more comprehensive strain resource for developing high-efficiency plant growth-promoting rhizobacteria.

Building upon the demonstrated potential of the isolate CHHM-1 and the existing limitation that current *P. syringae* pv. *actinidiae* control relies predominantly on prevention while lacking effective therapies for infected plants, future research should prioritize several strategic directions. These include the following key areas: First, enhancing the exploration of therapeutic biocontrol resources. This includes screening for biocontrol strains capable of invading the plant vascular system or inhibiting established pathogens through highly efficient antimicrobial substances. Second, advancing the development and application of novel antibiotics. It is recommended to screen for new antibiotic substances with specific inhibitory activity against *P. syringae* pv. *actinidiae* from the metabolites of biocontrol microorganisms. Third, developing combination strategies. This involves the scientific formulation of antibiotics with different mechanisms of action, low-dose copper-based agents, and biocontrol strains. Such integrated approaches could not only improve control efficacy but also effectively delay the development of *P. syringae* pv. *actinidiae* resistance.

In summary, this study successfully isolated and characterized *P. koreensis* CHHM-1 from the rhizosphere of *A. arguta* using a combination of dilution plating and a dual-culture plate assay against *P. syringae* pv. *actinidiae*, demonstrating significant antagonistic activity against *P. syringae* pv. *actinidiae*. The isolate CHHM-1 exhibits notable bactericidal activity against *P. syringae* pv. *actinidiae* through the secretion of bioactive compounds. The strain combines plant growth-promoting properties with environmental adaptability advantages. These findings establish the isolate CHHM-1 as a promising candidate for novel bioformulations that integrate disease control and growth enhancement, offering a sustainable solution for bacterial canker management.

## Figures and Tables

**Table 1:** Physiological and biochemical characteristics of the isolate CHHM-1.

Test	Result	Test	Test
Voges–Proskauer (VP)	−	Hydrogen sulfide	−
Indole production	−	Nitrate reduction	−
Gelatin liquefaction	+	Urease activity	−
Oxidase activity	+	Glucose utilization	+
Growth at pH 5.7	+	Starch hydrolysis	−

Note: Phenotypic characterization was performed using commercial biochemical test kits from Hopebio (*Bacillus cereus* identification kit and Gram-negative bacteria identification kit), based on the detection of enzymatic activities and metabolic responses to specific substrates. +, positive reaction; −, negative reaction.

**Table 2:** ANI analysis between the isolate CHHM-1 and the closest type strains of *Pseudomonas*.

Genome	16SrRNA Gene Sequence Similarity (%)	ANI (%)
*Pseudomonas koreensis* GCF_900101415.1	100	98.12
*P. putida* GCF_004683905.1	100	92.35
*P. fluorescens* GCF_000292795.1	99.61	92.26
*P. kribbensis* GCF_003352185.1	99.61	89.12
*P. moraviensis* GCF_900105805.1	99.80	88.24
*P. granadensis* GCF_900105485.1	99.35	88.19
*P. jessenii* GCF_002236115.1	99.61	86.34
*P. reinekei* GCF_001945365.1	99.61	86.14
*P. chlororaphis* GCF_001023535.1	99.61	84.62

Note: The genome contains the species name and Reference ID. 16S rRNA gene sequence similarity (%): percentage of identical bases in the 16S rRNA gene alignment; higher values indicate closer species relatedness. ANI (%): average nucleotide identity across the whole genome; higher values indicate closer genomic relatedness.

**Table 3:** Comparison of genomic components of the *P. koreensis*.

	CHHM-1	BM06	BS3658	P19E3	CRS05-R5	FP1691
origin	Liaoning	Nanjing	DOE Joint Genome Institute	Boppelsen	Hangzhou	Guangxi
Genome size (bp)	6,181,985	6,093,942	6,123,913	7,498,197	5,991,225	6,304,592
Number of chromosomes	1	1	1	5	1	1
GC percent (%)	60.5	60.5	60.5	59.0	60.5	60.0
Genome coverage	80.48×	318.4×	112×	159×	180×	219×

**Table 4:** Antibiotic resistance genes identified in *P. koreensis* CHHM-1 through CARD analysis.

ARO Name	ARO Accession	Resistance Mechanism	Identity (%)	Coverage (%)
*Pseudomonas* mutant *PhoP* conferring resistance to colistin	ARO:3003895	antibiotic efflux; antibiotic target alteration; resistance by absence	83.9	99.1
*Pseudomonas aeruginosa CpxR*	ARO:3004054	antibiotic efflux	82.6	99.1
*MexT*	ARO:3000814	antibiotic efflux	84.4	86.7
*MexW*	ARO:3003031	antibiotic efflux	82.6	99.4
*Pseudomonas aeruginosa parE* conferring resistance to fluoroquinolones	ARO:3003685	antibiotic target alteration	89.3	98.7
*MexF*	ARO:3000804	antibiotic efflux	87.9	99.2
*rsmA*	ARO:3005069	antibiotic efflux	80.6	98.4
*MexK*	ARO:3003693	antibiotic efflux	81.9	99.8
*MexB*	ARO:3000378	antibiotic efflux	80.3	99.2
*YajC*	ARO:3005040	antibiotic efflux	84.8	99.1
*Pseudomonas aeruginosa gyrA* and *parC* conferring resistance to fluoroquinolones	ARO:3003702	antibiotic target alteration	82.0	99.5

## Data Availability

The original contributions presented in this study are included in the article/Supplementary Materials. Further inquiries can be directed to the corresponding authors.

## Supplementary Materials

The following supporting information can be downloaded at https://www.mdpi.com/article/10.3390/microorganisms13102400/s1, Figure S1: In vitro antagonistic activity of *P. koreensis* CHHM-1 against the kiwifruit canker pathogen *P. syringae* pv. *actinidiae*. The assay was performed using a dual-culture plate method. A suspension of *P. syringae* pv. *actinidiae* (approximately 7 × 10^7^ CFU/mL) was diluted 10^4^-fold and spread evenly on a 15 cm NA medium. A sterile filter paper disc was placed in the center of the plate. (A) Control group: The disc was loaded with 10 μL of sterile water. No zone of inhibition was observed, indicating the disc itself had no inhibitory effect. (B) CHHM-1 treatment group: The disc was loaded with 10 μL of *P. koreensis* CHHM-1 culture. A distinct inhibition zone (average diameter of 4.81 cm) demonstrates the strong antagonistic activity of the isolate CHHM-1. The large central colony corresponds to the growth of *P. koreensis* CHHM-1 originating from the filter disc inoculum; Figure S2: Growth promotion effect of *P. koreensis* CHHM-1 inoculation on *A. arguta* seedlings. (A) A three-month-old *A. arguta* seedling following root immersion in the bacterial suspension of *P. koreensis* CHHM-1 for 2 min. (B) A control seedling of the same age treated with sterile water using an identical procedure. Both groups were cultivated under the same conditions for 7 days post-inoculation. Compared to the control, the isolate CHHM-1 inoculation significantly promoted plant growth, as evidenced by increases in fresh biomass (32.1–68.3%) and leaf number (45.3–75.1%). No significant difference in plant height was observed between the two groups; Figure S3: Assessment of the antifungal spectrum of the isolate CHHM-1 against four phytopathogenic fungi. The antagonistic activity was evaluated using a dual-culture assay on PDA (Hope Bio, Qingdao, China) plates. A mycelial plug of the test pathogen was placed in the center of the plate, flanked by two sterile 10 mm filter paper discs. For each panel, the left plate corresponds to the control group (disc loaded with 10 μL sterile water), and the right plate corresponds to the treatment group (disc loaded with 10 μL isolate CHHM-1 culture). (A) *Fusarium solani*: Both the control and the test discs were completely overgrown by the pathogen, indicating no inhibition. (B) *F. graminearum*: Both plates showed complete overgrowth with no zone of inhibition around either disc. (C) *F. oxysporum*: No antagonistic activity was observed, as evidenced by the full overgrowth of both plates. (D) *Ilyonectria robusta*: Similar to the other pathogens, the fungus fully colonized both plates, demonstrating the lack of inhibitory effect from the isolate CHHM-1. These results collectively indicate that the antifungal compound produced by the isolate CHHM-1 exhibits no significant inhibitory activity against these four fungal pathogens; Table S1: Antibiotic resistance genes identified in *P. koreensis* CHHM-1 through CARD database analysis.

## References

[1] Wang Y., Zhao C.L., Li J.Y., Liang Y.J., Yang R.Q., Liu J.Y., Ma Z., Wu L. (2018). Evaluation of biochemical components and antioxidant capacity of different kiwifruit (*Actinidia* spp.) genotypes grown in China. Biotechnol. Biotechnol. Equip..

[2] Wang Y., Liu Y. (2024). Recent advances of kiwifruit genome and genetic transformation. Mol. Hortic..

[3] Li D.W., Huang W.J., Zhong C.H. (2024). Current status of China’s kiwifruit industry and development recommendations for the 15th five-year plan. J. Fruit Sci..

[4] Dong H.T., Wu H.F., Shan L.L., Li R.N., Liu D.M. (2024). Variation characteristics and disaster risk analysis of strong wind in ripening period of *Actinidia arguta*: A case study of dandong area. Chin. Agric. Sci. Bull..

[5] Qin H.Y., Zhao Y., Chen X.L., Zhang B.X., Wen X., Li C.Y., Fan S.T., Wang Y., Yang Y.M., Xu P.L. (2023). Pathogens identification and resistance evaluation on bacterial canker in *Actinidia arguta* germplasm. J. Plant Pathol..

[6] He W.P., Liu W., Liu W.C., Huang L.L., Qin H.Q. (2025). Establishment of sensitivity baseline and resistance risk analysis of kiwifruit canker fungus for kasugamycin. Acta Agric. Boreali-Occident. Sin..

[7] Pereira C., Costa P., Pinheiro L., Balcão V.M., Almeida A. (2021). Kiwifruit bacterial canker: An integrative view focused on biocontrol strategies. Planta.

[8] Usuki G., Ishiga T., Sakata N., Ishiga Y. (2024). Flagellar motility of *Pseudomonas syringae* pv. *actinidiae* biovar 3 contributes to bacterial infection through stomata. J. Gen. Plant Pathol..

[9] Hu R., Xu X.H., Jia Y.J., Zhu C.C., Wang L., Song M.X., Xu Q., Xia M., He X.Q., Jin Y. (2025). Phage cocktail alleviates bacterial canker of kiwifruit by modulating bacterial community structure in field trial. Microorganisms.

[10] Guo Q., Shi M.D., Chen L., Zhou J.H., Zhang L.X., Li Y.L., Xue Q.H., Lai H.X. (2020). The biocontrol agent *Streptomyces pactum* increases *Pseudomonas koreensis* populations in the rhizosphere by enhancing chemotaxis and biofilm formation. Soil Biol. Biochem..

[11] De La Fuente L., Thomashow L., Weller D., Bajsa N., Quagliotto L., Chernin L., Arias A. (2004). *Pseudomonas fluorescens* UP61 isolated from birdsfoot trefoil rhizosphere produces multiple antibiotics and exerts a broad spectrum of biocontrol activity. Eur. J. Plant Pathol..

[12] Wang B.C., Guo Y.S., Chen X.T., Ma J.L., Xia L., Wang W.Z., Long Y.H. (2023). Assessment of the biocontrol potential of *Bacillus velezensis* WL–23 against kiwifruit canker caused by *Pseudomonas syringae* pv. *actinidiae*. Int. J. Mol. Sci..

[13] Saitou N., Nei M. (1987). The neighbor-joining method: A new method for reconstructing phylogenetic trees. Mol. Biol. Evol..

[14] Zhu H.X., Hu L.F., Hu H.Y., Zhou F., Wu L.L., Wang S.W., Rozhkova T., Li C.W. (2023). Identification of a novel *Streptomyces* sp. strain HU2014 showing growth promotion and biocontrol effect against *Rhizoctonia* spp. in wheat. Plant Dis..

[15] Pang J., Liu Y.M., Huang Y.C., Wang C.R., Liu B., Liu Z.Q., Huang Y.Z., Huang Y.F., Zhang C.B. (2021). Isolation and identification of the plant endophyte R13 and its effect on cadmium accumulation in *Solanum nigrum* L.. Huan Jing Ke Xue.

[16] Meng C.N., Zhao Y.J., Chen J.X., Zhang Y.L., Wang Y.J., Feng L.R., Sun Y.G., Guo C.H. (2024). Screening and identification of two strains of nitrogen-fixing bacteria from the silage maize rhizosphere and their roles in plant growth promotion. Acta Prataculturae Sin..

[17] Yu H.X., Liang H.L., Wang Z.X., Yang X.Y., Li W.H. (2022). Isolation, identification and growth-promoting effects of culturable nitrogen-fixing bacteria and ammonifying bacteria in rhizosphere soil of *Mikania micrantha*. Acta Microbiol. Sin..

[18] Zhao Z.B., Chen J.L., Gao X.N., Zhang D., Zhang J.L., Wen J., Qin H.Q., Guo M., Huang L.L. (2019). Comparative genomics reveal pathogenicity-related loci in *Pseudomonas syringae* pv. *actinidiae* biovar 3. Mol. Plant Pathol..

[19] Tian R.Z., Tian Y.J., Mi Q.Q., Huang L.L. (2025). Histocytological analysis reveals the biocontrol activity of a rhizospheric bacterium *Pseudomonas rhizophila* Z98 against kiwifruit bacterial canker. Pestic. Biochem. Physiol..

[20] Yan Z.W., Fu M., Mir S.H., Zhang L.X. (2023). Diversity and characterization of antagonistic bacteria against *Pseudomonas syringae* pv. *actinidiae* isolated from kiwifruit rhizosphere. FEMS Microbiol. Lett..

[21] Zhu H.Y., Ma Y., Ke Y., Li B. (2021). Screening and identification of an antagonist against the pathogen of kiwifruit canker and its antifungal activity to the phytopathogenic fungus. Biotechnol. Bull..

[22] Ali M.A., Luo J.Y., Ahmed T., Zhang J.N., Xie T., Dai D.J., Jiang J.Y., Zhu J., Hassan S., Alorabi J. (2022). *Pseudomonas bijieensis* strain XL17 within the *P. corrugata* subgroup producing 2,4-diacetylphloroglucinol and lipopeptides controls bacterial canker and gray mold pathogens of kiwifruit. Microorganisms.

[23] Daranas N., Roselló G., Cabrefiga J., Donati I., Francés J., Badosa E., Spinelli F., Montesinos E., Bonaterra A. (2019). Biological control of bacterial plant diseases with *Lactobacillus plantarum* strains selected for their broad-spectrum activity. Ann. Appl. Biol..

[24] Zhao X., Zhai Y., Wei L., Xia F., Yang Y.R., Yi Y.J., Wang H.Y., Qiu C.S., Wang F., Zeng L.B. (2024). Isolation and identification of a novel *Bacillus velezensis* strain JIN4 and its potential for biocontrol of kiwifruit bacterial canker caused by *Pseudomonas syringae* pv. *actinidiae*. Front. Plant Sci..

[25] Ma J.T., Dong X.Y., Li Z.H., Yan H., He J., Liu J.K., Feng T. (2023). Antibacterial metabolites from kiwi endophytic fungus *Fusarium tricinctum*, a potential biocontrol strain for kiwi canker disease. J. Agric. Food Chem..

[26] Biondi E., Gallipoli L., Mazzaglia A., Fuentealba S.P., KuzmanoviĆ N., Bertaccini A., Balestra G. (2021). Bacillus-based products for management of kiwifruit bacterial canker. Phytopathol. Mediterr..

[27] Shao X., Wu Q., Li L., He W., He X., Cheng D., Murero A., Lin L., Wang L., Zhong C. (2024). Adapting the inoculation methods of kiwifruit canker disease to identify efficient biocontrol bacteria from branch microbiome. Mol. Plant Pathol..

[28] Ren Y.X., Zhou X.H., Cai B.Y., Sun Y.C., Ge J.P., Ping W.X. (2025). Effects of fermented polyphenols from *Lonicera caerulea* on *Streptococcus mutans* pathogenicity: Exopolysaccharide structure and quorum sensing regulation. Int. J. Biol. Macromol..

[29] Kaur M., Jangra M., Singh H., Tambat R., Singh N., Jachak S., Mishra S., Sharma C., Nandanwar H., Pinnaka A. (2019). *Pseudomonas koreensis* recovered from raw yak milk synthesizes a β-carboline derivative with antimicrobial properties. Front. Microbiol..

[30] Wei X.H., Nie Q.W., Medison R.G., Zheng T.W., Meng X.J., Sun Z.X., Zhou Y. (2024). Evaluation of *Pseudomonas koreensis* B17-12 as a potential biological control agent against postharvest diseases of tomato. Physiol. Mol. Plant Pathol..

[31] Vemic A., Jovanovic S., Beric T., Lucic A., Rakonjac L., Mitrovic S., Popovic V. (2025). The potential of *Pseudomonas koreensis* R4.45P to suppress *Hymenoscyphus fraxineus* development in *Fraxinus excelsior* leaves. For. Pathol..

[32] Ghazy N., El Nahrawy S. (2021). Siderophore production by *Bacillus subtilis* MF497446 and *Pseudomonas koreensis* MG209738 and their efficacy in controlling *Cephalosporium maydis* in maize plant. Arch. Microbiol..

[33] Chen T., Wang Y., Chi X.H., Xiong L.Y., Lu P., Wang X.T., Chen Y.B., Luo Q., Shen P., Xiao Y.H. (2024). Genetic, virulence, and antimicrobial resistance characteristics associated with distinct morphotypes in ST11 carbapenem-resistant *Klebsiella pneumoniae*. Virulence.

[34] Guo Q., Sun Y., Shi M., Han X., Jing Y., Li Y., Li H., Lai H. (2021). *Pseudomonas koreensis* promotes tomato growth and shows potential to induce stress tolerance via auxin and polyphenol-related pathways. Plant Soil.

[35] Kalleku J.N., Ihsan S., Al-Azzawi T.N.I., Khan M., Hussain A., Chebitok F., Das A.K., Moon Y., Mun B., Lee I. (2024). Halotolerant *Pseudomonas koreensis* S4T10 mitigate salt and drought stress in *Arabidopsis thaliana*. Physiol. Plant.

[36] Ali S., Khan M., Moon Y. (2025). Synergistic effect of *Serratia fonticola* and *Pseudomonas koreensis* on mitigating salt stress in *Cucumis sativus* L.. Curr. Issues Mol. Biol..

[37] Hkudaygulov G., Chetverikova D., Kendzieva A., Rameev T., Timergalin M., Feoktistova A., Starikov S., Sharipov D., Sultangazin Z., Chukbar N. (2021). Mitigating effect of PGP-bacteria *Pseudomonas koreensis* IB4 on growth and biochemical parameters of wheat plants during their treatment with herbicides. Biosci. Res..

[38] Vincze É., Becze A., Salamon R., Lányi S., Mara G. (2024). Role of the *Pseudomonas koreensis* BB2.A.1 and *Serratia liquefaciens* BB2.1.1 bacterial strains in maize trace metal stress management. Microorganisms.

