# Supplementary material for: Biocontrol Potential, Plant Growth-Promotion, and Genomic Insights of Pseudomonas koreensis CHHM-1 Against Bacterial Canker in Actinidia arguta
Source: Microorganisms. 2025 Oct 20;13(10):2400. doi: 10.3390/microorganisms13102400 (PMC12566068; doi:10.3390/microorganisms13102400)
Supplement: Supplementary file 1 [file microorganisms-13-02400-s001.zip › microorganisms-3895003-supplementary/Supplementary/Supplementary table.pdf]

Supplementary Table S1. Antibiotic resistance genes identified in *P. koreensis* CHHM-1 through CARD database analysis.

Gene ID	ARO Name	ARO Accession	ARO Description	Drug Class	Resistance Mechanism	Identity(%)	Coverage(%)	Evalue	Score
gene4014	*Pseudomonas* mutant *PhoP* conferring resistance to colistin	ARO:3003895	Mutations in *Pseudomonas aeruginosa PhoP* of the two-component *PhoPQ* regulatory system. Presence of mutation confers resistance to colistin.	macrolide antibiotic; peptide antibiotic	antibiotic efflux;antibiotic target alteration;resistance by absence	83.9	99.1	5.27E-135	964
gene1450	*Pseudomonas aeruginosa CpxR*	ARO:3004054	*CpxR* is directly involved in activation of expression of RND efflux pump *MexAB-OprM* in *P. aeruginosa. CpxR* is required to enhance *mexAB-oprM* expression and drug resistance, in the absence of repressor *MexR*.	aminocoumarin antibiotic;aminoglycoside antibiotic;carbapenem;cephalosporin;cephamycin;diaminopyrimidine antibiotic;fluoroquinolone antibiotic;macrolide antibiotic;monobactam;penam;penem;peptide antibiotic;phenicol antibiotic;sulfonamide antibiotic;tetracycline antibiotic	antibiotic efflux	82.6	99.1	4.82E-131	938

gene2798	*MexT*	ARO:3000814	*MexT* is a LysR-type transcriptional activator that positively regulates the expression of *MexEF-OprN*, *OprD*, and *MexS*.	diaminopyrimidine antibiotic;fluoroquinolone antibiotic;phenicol antibiotic	antibiotic efflux	84.4	86.7	1.32E-160	1154
gene4268	*MexW*	ARO:3003031	*MexW* is the RND-type membrane protein of the efflux complex *MexVW-OprM*.	disinfecting agents and antiseptics;fluoroquinolone antibiotic;macrolide antibiotic;phenicol antibiotic;tetracycline antibiotic	antibiotic efflux	82.6	99.4	0	4089
gene0485	*Pseudomonas aeruginosa parE* conferring resistance to fluoroquinolones	ARO:3003685	Point mutation in *Pseudomonas aeruginosa parE* resulting in sensitivity to fluoroquinolones (ciprofloxacin). In combination with a gyrase mutation (*gyrA* or *gyrB*), it confers a high level of resistance to ciprofloxacin.	fluoroquinolone antibiotic	antibiotic target alteration	89.3	98.7	0	2929
gene2806	*MexF*	ARO:3000804	*MexF* is the multidrug inner membrane transporter of the *MexEF-OprN* complex. *mexF* corresponds to 2 loci in *Pseudomonas aeruginosa*	diaminopyrimidine antibiotic;fluoroquinolone antibiotic;phenicol antibiotic	antibiotic efflux	87.9	99.2	0	4614

			PAO1 (gene name: *mexF/mexB*) and 4 loci in *Pseudomonas aeruginosa* LESB58 (gene name: *mexD/mexB*).						
gene4050	*rsmA*	ARO:3005069	*rsmA* is a gene that regulates virulence of *Pseudomonas aeruginosa*. However, its negative effect on *MexEF-OprN* overexpression has been noted to confer resistance to various antibiotics. It's *Escherichia coli* homolog is *csrA*.	diaminopyrimidine antibiotic;fluoroquinolone antibiotic;phenicol antibiotic	antibiotic efflux	80.6	98.4	5.62E-30	241
gene1060	*MexK*	ARO:3003693	*MexK* is the inner membrane resistance-nodulation-cell division (RND) transporter in the *MexJK* multidrug efflux protein.	disinfecting agents and antiseptics;macrolide antibiotic;tetracycline antibiotic	antibiotic efflux	81.9	99.8	0	4202
gene1262	*MexB*	ARO:3000378	*MexB* is the inner membrane multidrug exporter of the efflux complex *MexAB-OprM*.	aminocoumarin antibiotic;carbapenem;cephalosporin;cephamycin;diaminopyrimidine antibiotic;fluoroquinolone antibiotic;macrolide	antibiotic efflux	80.3	99.2	0	4174

				antibiotic;monobactam;penam;penem;peptide antibiotic;phenicol antibiotic;sulfonamide antibiotic;tetracycline antibiotic					
gene4400	*YajC*	ARO:3005040	*YajC* interacts with the AcrAB-TolC efflux pump in a way that in uncharacterized but is shown to grant increased fitness in the presence of linezolid, rifampicin, and vancomycin.	cephalosporin;disinfecting agents and antiseptics;fluoroquinolone antibiotic;glycopeptide antibiotic;glycylcycline;oxazolidinone antibiotic;penam;phenicol antibiotic;rifamycin antibiotic;tetracycline antibiotic	antibiotic efflux	84.8	99.1	3.98 E-64	475
gene0488	*Pseudomonas aeruginosa gyrA* and *parC* conferring resistance to fluoroquinolones	ARO:3003702	Point mutation in *Pseudomonas aeruginosa parC* resulting in fluoroquinolone resistance also requiring a *gyrA* mutation.	fluoroquinolone antibiotic	antibiotic target alteration	82	99.5	0	3146
