# Supplementary figures and images for: Biocontrol Potential, Plant Growth-Promotion, and Genomic Insights of Pseudomonas koreensis CHHM-1 Against Bacterial Canker in Actinidia arguta
Source: Microorganisms. 2025 Oct 20;13(10):2400. doi: 10.3390/microorganisms13102400 (PMC12566068; doi:10.3390/microorganisms13102400)

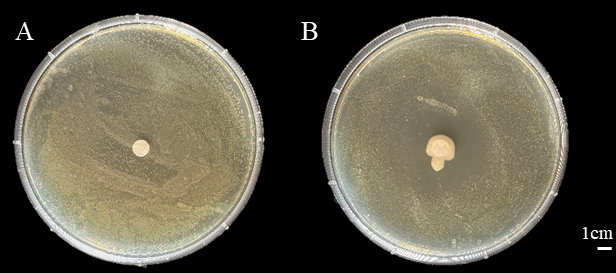

Supplement: Supplementary file 1 [file microorganisms-13-02400-s001.zip › microorganisms-3895003-supplementary/Supplementary/Supplementary Figure/Supplementary Figure S1.png]

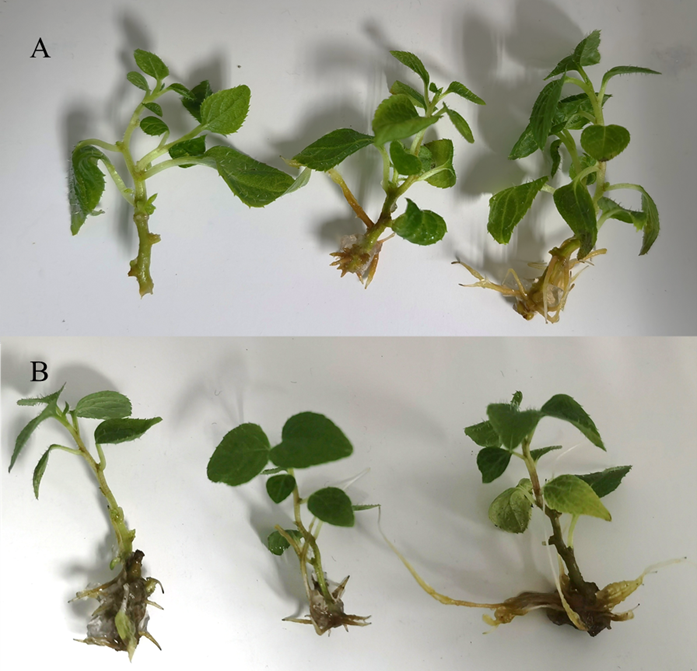

Supplement: Supplementary file 1 [file microorganisms-13-02400-s001.zip › microorganisms-3895003-supplementary/Supplementary/Supplementary Figure/Supplementary Figure S2.png]

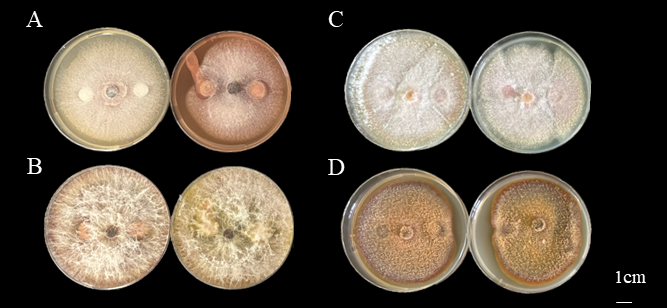

Supplement: Supplementary file 1 [file microorganisms-13-02400-s001.zip › microorganisms-3895003-supplementary/Supplementary/Supplementary Figure/Supplementary Figure S3.png]
